# Clinical and Bacteriological Analysis of Pediatric Pneumococcal Meningitis after 13-Valent Pneumococcal Conjugate Vaccine Introduction in Japan

**DOI:** 10.1128/spectrum.01822-21

**Published:** 2022-03-31

**Authors:** Erika Kurihara, Kenichi Takeshita, Saori Tanaka, Noriko Takeuchi, Misako Ohkusu, Haruka Hishiki, Naruhiko Ishiwada

**Affiliations:** a Department of Pediatrics, Chiba Kaihin Municipal Hospital, Chiba, Japan; b Department of Pediatrics, Chiba University Hospital, Chiba, Japan; c Department of Pediatrics, Chiba Rosai Hospital, Chiba, Japan; d Department of Infectious Diseases, Medical Mycology Research Center, Chiba University, Chiba, Japan; Quest Diagnostics Nichols Institute

**Keywords:** *Streptococcus pneumoniae*, meningitis, serotype, antimicrobial susceptibility, 13-valent pneumococcal conjugate vaccine

## Abstract

Streptococcus pneumoniae is one of the leading causes of meningitis in children. In Japan, since the introduction of the 13-valent pneumococcal conjugate vaccine (PCV13), the number of pneumococcal meningitis due to non-PCV13 serotypes in children has increased. To clarify the clinical outcomes, serotype distributions, and antimicrobial susceptibility of isolated S. pneumoniae strains from pediatric pneumococcal meningitis, we clinically and bacteriologically analyzed 34 cases of pediatric pneumococcal meningitis that were reported after the PCV13 introduction era in Japan. The median age at diagnosis was 1 year (range: 3 months–13 years). Ten (29.4%) patients had underlying diseases. Twenty-nine (85.3%) patients had received at least one dose of any pneumococcal vaccine. Of the 34 patients with pneumococcal meningitis, 6 had sequelae, and 4 died. Nine (26.5%) strains were resistant to penicillin; five (15%) strains to meropenem, with an MIC of 0.5 μg/mL. All strains were susceptible to vancomycin and linezolid. Daptomycin’s MIC_50_ was 0.064 μg/mL and MIC_90_ was 0.094 μg/mL. Among the tested strains, only four were PCV13 serotypes. Penicillin-resistant S. pneumoniae was isolated from 30.0% of the patients with sequelae and death. Particularly, the proportion of serotype 10A in the sequelae and deceased cases was significantly higher than that in the complete recovery cases. We should carefully monitor the serotype and drug susceptibility of S. pneumoniae strains isolated from patients with meningitis after the PCV13 era and reconsider the treatment strategy to prepare against further drug-resistant pneumococcal strains.

**IMPORTANCE** We analyzed 34 cases of pediatric pneumococcal meningitis that were reported after the 13-valent pneumococcal conjugate vaccine (PCV13) introduction era in Japan. Our study revealed that pneumococcal meningitis in children was mainly caused by non-PCV13 serotypes; all cases with sequelae and death were caused by non-PCV13 serotypes. Moreover, all serotypes of penicillin resistant Streptococcus pneumoniae strains (26.5%; 9/34) were non-PCV13 serotypes. We also analyzed antimicrobial susceptibilities of glycopeptides, linezolid (LZD), and daptomycin (DAP) of isolated S. pneumoniae strains. All tested strains were susceptible to vancomycin, teicoplanin, LZD, and DAP. Especially. DAP demonstrated the best outcome among the tested antibiotics, with MIC_90_ of 0.094 μg/mL. Pneumococcal meningitis in children continues to persist and is difficult to control with the current conjugate vaccines. Therefore, it is important to monitor the serotype and antimicrobial susceptibility of S. pneumoniae strains isolated from patients with meningitis and accordingly reconsider the treatment strategy.

## INTRODUCTION

Streptococcus pneumoniae is frequently isolated from the nasopharynx of children and is an important cause of respiratory infectious diseases, such as bronchitis, pneumonia, otitis media, and sinusitis. S. pneumoniae also induces invasive pneumococcal diseases (IPDs), such as bacteremia and meningitis.

To prevent IPD, the heptavalent-pneumococcal conjugate vaccine (PCV7) was introduced as a voluntary immunization in Japan, following which it was included in the routine immunization program in Japan in April 2013. By November 2013, 13-valent pneumococcal conjugate vaccine (PCV13) replaced it in the routine immunization program. In Japan, after the introduction of PCV7 and its replacement by PCV13, the number of reported cases of pneumococcal meningitis in children decreased drastically; however, the amount of pneumococcal meningitis due to nonvaccine type has increased (1, 2). Further, the incidence rate of pediatric pneumococcal meningitis due to non-PCV13 serotypes has increased after the introduction of PCV13 in various regions worldwide (3–5).

Pneumococcal meningitis is associated with a high mortality rate. Although the illness is treated, severe sequelae often persist. Therefore, early diagnosis and appropriate treatment of pneumococcal meningitis are important. S. pneumoniae still has been the second most common pathogen of pediatric bacterial meningitis after Streptococcus agalactiae in Japan since 2012 (2). Furthermore, IPDs caused by penicillin, cephalosporin, and macrolide-resistant S. pneumoniae have been reported in Japan (6, 7). Meningitis caused by meropenem (MEPM)-resistant non-PCV13 serotypes *of* S. pneumoniae was recently reported (8). Based on the Japanese guidelines for bacterial meningitis, cefotaxime (CTX) or ceftriaxone (CTRX) plus vancomycin (VCM) is recommended as an empirical therapy for bacterial meningitis because it covers penicillin-resistant S. pneumoniae (PRSP) (9). In the light of current knowledge, new therapeutic options must be investigated for pneumococcal meningitis caused by multidrug-resistant S. pneumoniae.

In this study, we analyzed the clinical outcomes, serotypes, and antimicrobial susceptibility testing of glycopeptides, linezolid (LZD), and daptomycin (DAP) of isolated S. pneumoniae strains from pediatric pneumococcal meningitis after the PCV13 era in Japan. This study aimed to clarify the current situation of pneumococcal meningitis in children after the PCV13 era in Japan and to investigate new therapeutic options for pneumococcal meningitis.

## RESULTS

### Patients’ characteristics.

During the study period, 34 episodes of pneumococcal meningitis were observed. Table 1 presents the patients’ characteristics in this study. Of the 34 episodes, 14 were experienced in Chiba Prefecture and 20 were experienced outside Chiba Prefecture; 10 were in Tokyo, 2 were in Miyagi Prefecture, 2 were in Hyogo Prefecture, and 1 each in Nagasaki, Kanagawa, Osaka, Shizuoka, Gunma, and Oita Prefectures. Thus, Chiba Prefecture and Tokyo accounted for a large proportion of the total patients, and samples were collected from all over Japan (12/47 prefectures). The median age at diagnosis was 1 year (range: 3 months–13 years). Of the 34 episodes, 50.0% (*n* = 17) were experienced by males and 50.0% (*n* = 17) were experienced by females. Ten patients had underlying diseases, including two cases of hereditary spherocytosis and one case each of Wiskott-Aldrich syndrome, acute myeloid leukemia, Ohtahara syndrome, trisomy 21, hydrocephalus and mental retardation, congenital cholesteatoma, deafness, and cleft lip.

**TABLE 1:** Patient’s characteristics in this study (*n* = 34)^*a*^

Characteristic	Variable	Number (%) of the patients
Age	<6 mo 6–23 mo 2–4 yrs 5–9 yrs 10–13 yrs	4 (11.8%) 17 (50.0%) 3 (8.8%) 6 (17.6%) 4 (11.8%)
Sex	Male Female	17 (50.0%) 17 (50.0%)
Underlying disease	Yes No	10 (29.4%) 24 (70.6%)
Vaccine history	PCV7 PCV13 PPSV23 None	6 (17.6%) 21 (61.8%) 2 (5.9%) 5 (14.7%)
Serotype of isolated strain	PCV13 serotypes PPSV23-nonPCV13 serotypes Non-vaccine serotypes	4 (11.8%) 14 (41.2%) 16 (47.0%)

a PCV7, heptavalent-pneumococcal conjugate vaccine; PCV13, 13-valent pneumococcal conjugate vaccine; PPSV23, 23-valent pneumococcal polysaccharide vaccine.

Of the 34 patients, 5 (14.7%) were unvaccinated and 29 (85.3%) had received at least one dose of PCV or 23-valent pneumococcal polysaccharide vaccine (PPSV23). Of the 29 vaccinated patients, 6 received PCV7, 21 received PCV13, and 2 with hereditary spherocytosis as an underlying disease received PPSV23. Of the five patients with no vaccination history, four were outside the PCV target age, and one patient was 10 months old.

### Antimicrobial susceptibility testing of isolated strains.

Table 2 shows the results of the antimicrobial susceptibility testing. For TEIC and DAP, there were no data that clearly indicated the breakpoints; therefore, only the MIC values are shown.

**TABLE 2:** MIC ranges of antimicrobial susceptibility measurement^*a*^

Name of drug	MIC ranges (μg/mL)	MIC_50_ (μg/mL)	MIC_90_ (μg/mL)
PCG	≤0.015–1	0.03	0.5
ABPC	≤0.03–4	0.06	2
CTX	≤0.03–1	0.25	0.5
CTRX	≤0.03–1	0.25	0.5
MEPM	≤0.008–0.5	0.015	0.5
AZM	0.12–>8	>8	>8
CLDM	≤0.12–>4	>4	>4
EM	≤0.12–>4	>4	>4
VCM	≤0.12–0.5	0.25	0.5
LZD	0.25–1	0.5	1
TEIC	≤0.25	≤0.25	≤0.25
DAP	0.023–0.19	0.064	0.094

a MIC, minimum inhibitory concentrations; PCG, penicillin G; ABPC, ampicillin; CTX, cefotaxime; CTRX, ceftriaxone; MEPM, meropenem; AZM, azithromycin; CLDM, clindamycin; EM, erythromycin; VCM, vancomycin; LZD, linezolid; TEIC, teicoplanin; DAP, daptomycin. Antimicrobial susceptibility testing for DAP was performed by the Etest. Antimicrobial susceptibility testing for the drugs, except DAP, was performed by broth microdilution.

The MIC_90_ and MIC_50_ of PCG were 0.5 μg/mL and 0.03 μg/mL, respectively. Among the 34 strains, nine (26.5%) were PRSP, with an MIC of ≥0.12 μg/mL. All strains were susceptible to VCM and LZD. The MIC_90_ of MEPM was 0.5 μg/mL, and 15% (5/34) of the strains showed resistance to MEPM. The MIC_90_ of EM were >4 μg/mL. All the strains of MICs of TEIC were found to be ≤0.25 μg/mL. The MIC_50_ of DAP was 0.064 μg/mL, and the MIC_90_ of DAP was 0.094 μg/mL.

### Serotype of isolated strains.

**FIG 1:**
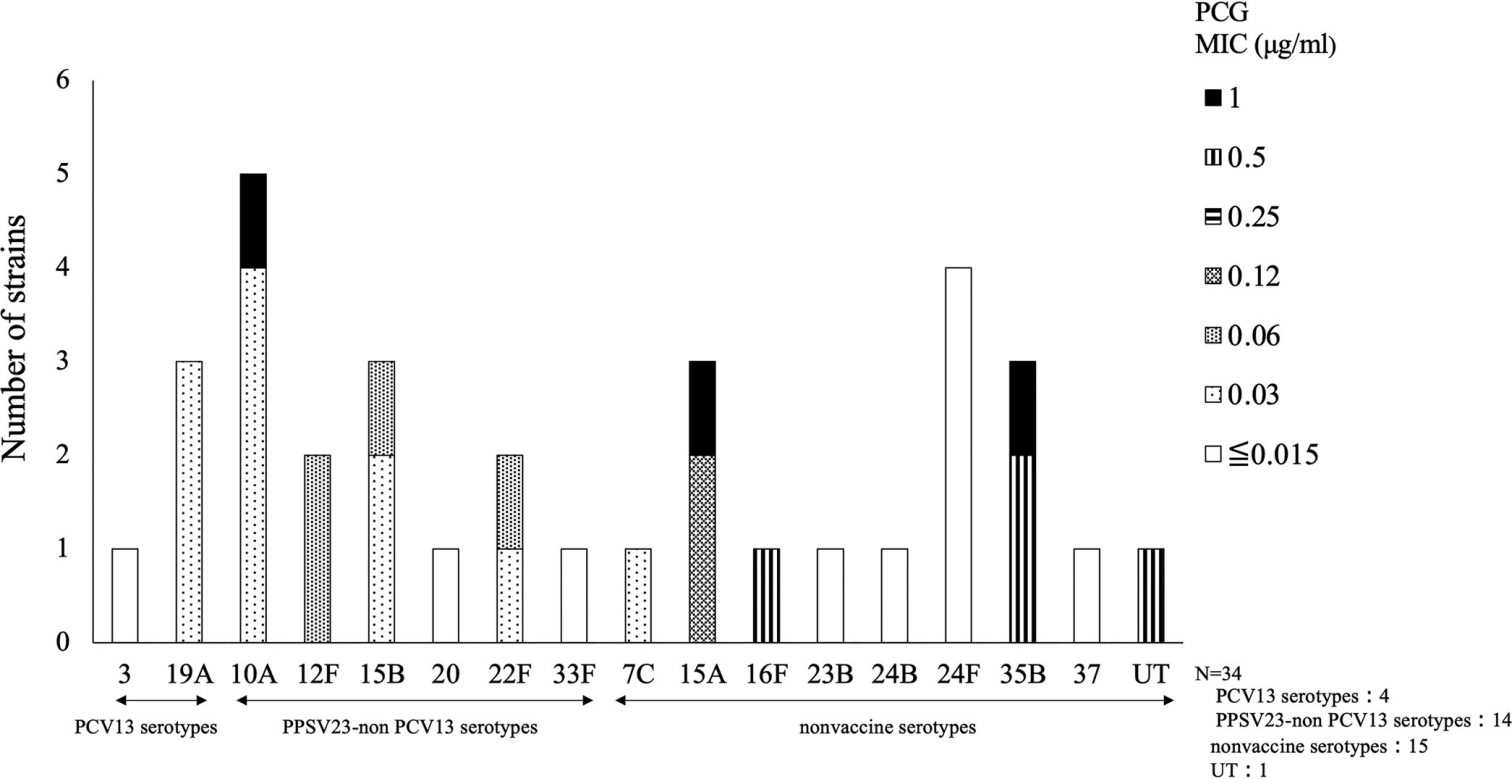
The serotype distribution of the 34 strains and their MIC of penicillin G. PCG, penicillin G; MIC, minimum inhibitory concentrations; PCV13, 13-valent pneumococcal conjugate vaccine; PPSV23, 23-valent pneumococcal polysaccharide vaccine; UT, untypeable.

Fig. 1 shows the serotypes of the 34 strains and their PCG MICs. Four strains were PCV13 serotypes (19A: 3 strains; 3: 1 strain), 14 strains were PPSV23-nonPCV13 serotypes (10A: 5 strains; 15B: 3 strains; 12F and 22F: 2 strains each; 20 and 33F: 1 strain each), 15 strains were nonvaccine serotypes (24F: 4 strains; 15A and 35B: 3 strains each; 7C, 16F, 23B, 24B, and 37; 1 strain each), and 1 strain was untypeable. The untypeable strain was a nonencapsulated S. pneumoniae. In summary, 18 (52.9%) strains were PCV13/PPSV23 serotypes, and 16 (47.0%) strains were non PCV13/PPSV23 serotypes. In terms of antimicrobial susceptibility, 26.5% (9/34) of the strains were PRSP with a PCG MIC of ≥0.12 μg/mL. The serotypes of PRSP strains were 15A (3 strains), 35B (3 strains), 10A (1 strain), 16F (1 strain), and untypeable (1 strain). All PRSP strains were non-PCV13 serotypes.

### Clinical outcomes.

Of the 34 episodes, 24 showed complete recovery, 6 had sequelae, and 4 resulted in death. The sequelae included various symptoms, such as temporary epilepsy and severe mental and physical disabilities. Two patients became deaf. Of the four patients who died, one was unvaccinated because of overage for the PCV vaccination, and three had received PCV13. PRSP was isolated from 25.0% of patients with complete recovery and 30.0% of patients with sequelae and death.

We performed a statistical analysis of clinical outcomes (Table 3). There were more cases of sequelae and death in younger age groups, but there was no significant difference between patients younger than 2 years old (*P* = 0.16) and younger than 5 years old (*P* = 0.44). There was no significant difference in sequelae or death between patients with and without underlying diseases (*P* = 0.96). Serotypes of the strain isolated from the case with sequelae were 10A (2 strains), and one each of 7C, 20, 24F, and 35B. The serotypes of the four strains isolated from the fatal cases were 10A (2 strains), 15A (1 strain), and 15B (1 strain). The proportion of serotype 10A in the sequelae and deceased cases was significantly higher than that in the complete recovery cases (*P* = 0.019, performed by Fisher’s exact test). PCV13 serotypes were not isolated from cases with sequelae or deceased cases. PPSV23-nonPCV13 serotypes had a higher isolation rate in cases with sequelae and deceased cases compared with other serotypes, but the rate was not significantly different (*P* = 0.15). The prognosis was not statistically different between meningitis caused by PCV13/PPSV23 serotypes and that caused by nonvaccine serotypes.

**TABLE 3:** Statistical analysis of clinical outcomes using the chi-squared test (*n* = 34)^*a*^

Probale factors related to outcome	Variable	Complete recovery	Sequelae + (death)	*P* value
Age	<2y ≥2y	13 11	8 (3) 2 (1)	0.16
Underlying disease	Yes No	7 17	3 (1) 7 (3)	0.96
Vaccination history of PCV13	Yes No	13 11	8 (3) 2 (1)	0.16
Serotype of isolated strain	10A Non-10A	1 23	4 6	0.019
	PPSV23-nonPCV13 Nonvaccine serotypes	8 16	6 4	0.15
Penicillin resistance of isolated strain	PRSP Non-PRSP	6 18	3 (1) 7 (3)	0.76

a PCV13, 13-valent pneumococcal conjugate vaccine; PPSV23, 23-valent pneumococcal polysaccharide vaccine; PRSP, penicillin-resistant S. pneumoniae.

## DISCUSSION

In countries with a mature vaccination program of PCV, the proportion of pneumococcal meningitis caused by vaccine serotypes is lower than that which was seen in the pre-PCV era (10). In this study, among the 34 episodes of pneumococcal meningitis after PCV13 introduction, most episodes (30/34) were caused by non-PCV13 serotypes. In addition, there were two or more isolates of serotypes 10A, 12F, 15A, 15B, 22F, 24F, and 35B in this study. In terms of serotype distribution of pediatric pneumococcal meningitis in Japan, Shinjoh et al. also reported that the proportion of serotypes 15A, 15C, 24F, and 35B were significantly higher in 2014–2016 compared to the pre-PCV13 period in 2010 (2).

According to a study from France, the most frequent non-PCV13 emergent serotypes were 10A, 12F, 15A, 15B, 15C, 22F, 23B, and 24F after the introduction of PCV13. Furthermore, the proportion of serotype 24F penicillin-resistant isolates increased from the early PCV13 period to the late PCV13 period (3). In a study from England and Wales, during the PCV13 period, after adjusting for age and year of diagnosis, the odds of meningitis were higher for serotypes 10A, 22F, 23B, and 35B (4). On the other hand, in a study from Mexico, PCV13 reduced pneumococcal meningitis and the disappearance of serotype 19A (11). Nevertheless, in our study, serotype 19A remained one of the causative serotypes after PCV13 introduction.

After the introduction of PCV7 in Japan, the rate of PRSP among the causative S. pneumoniae of IPD decreased. Moreover, Ubukata et al. have reported that genotypic penicillin resistance decreased from 54.3% in 2010 to 11.2% in 2016 (12). In our study, the PRSP rate was 26.5% (9/34). Recently, various countries have reported on the rate of PRSP among isolated strains of pediatric pneumococcal meningitis. According to these reports, the rates of PRSP in Niger and Israel were 16% and 26.6%, respectively (5, 13). After the introduction of PCV, PRSP still accounts for approximately 30% of the causative S. pneumoniae in pediatric meningitis cases worldwide. In our study, serotypes 10A, 15A, 16F, and 35B and nonencapsulated strain were PRSP. Serotypes 15A and 35B increased after PCV13 introduction in various countries, including Japan (14–16). These two serotypes are well known for their multidrug resistance (7, 8, 16).

Bacterial meningitis is a life-threatening infectious disease that often manifests as a severe condition. In our study, 29.4% (10/34) of the patients had a poor prognosis. Patient’s age and underlying disease were not related to prognosis. All cases with poor prognosis were caused by non-PCV13 serotypes. Regardless of the PCV13 introduction era, we continue to keep in mind the early diagnosis and appropriate treatment for pneumococcal meningitis. In terms of the association between serotype and prognosis, serotype 10A had a relatively higher rate of poor diagnosis. Severe pneumococcal meningitis caused by serotype 10A has also been reported (17). Among the nine patients with meningitis caused by PRSP, including serotype 10A, one patient was deceased, and two patients had severe sequelae (epilepsy and hearing loss).

CTRX plus VCM is recommended for PRSP meningitis. However, CSF VCM levels are variable and cannot predict clinical cure. VCM penetrates the CSF, requires therapeutic drug monitoring, and is occasionally complicated by side effects such as Redman syndrome or renal disturbance. Effective antibiotics against methicillin-resistant Staphylococcus aureus (MRSA) are also expected to be effective against PRSP. Therefore, the candidate antibiotics for the treatment of PRSP other than VCM are listed as TEIC, LZD, and DAP. However, information on the sensitivity of these antimicrobial agents against PRSP is limited. In our study, all tested strains were susceptible to VCM and LZD.

According to a report evaluating the role of LZD in the treatment of pneumococcal meningitis, as an alternative to VCM (18), treatment for bacterial meningitis requires high-dose and long-term antibiotic use. Therefore, the use of LZD for bacterial meningitis requires careful observation due to side effects such as neutropenia and digestive symptoms.

There are no reports on the efficacy of TEIC and DAP for the treatment of pneumococcal meningitis in children. TEIC has a long half-life, and all tested strains in this study had low MICs. However, according to a report on treatment of adult MRSA meningitis, clinical efficacy was not expected, except in a few reports (19). In terms of drug susceptibility of DAP against S. pneumoniae in this study, MIC_50_ and MIC_90_ were 0.064 and 0.094 μg/mL, respectively. Compared with the CLSI breakpoint criteria against MRSA (S: MIC ≤1 μg/mL), the drug susceptibility of DAP against S. pneumoniae is good. There are several reports on the efficacy of DAP against pneumococcal meningitis in animal models. Vivas et al. have reported that the penetration of DAP into the CSF ranged from 9% to 11% in penicillin- and cephalosporin-resistant rabbit pneumococcal meningitis models. They concluded that high-dose DAP therapy may be a useful alternative for the treatment of drug-resistant pneumococcal meningitis (20). Another study has also reported that DAP and CTRX combination therapy is effective in experimental infant rat pneumococcal meningitis (21).These reports and the drug susceptibility reported in our study supported the necessity of the alternative use of DAP against PRSP meningitis in situations where VCM cannot be easily used.

After the PCV13 era, the causative serotypes of pneumococcal meningitis changed from mainly PCV13 serotypes to non-PCV13 serotypes, including PRSP. In the U.S., a 20-valent pneumococcal conjugate vaccine (PCV20) containing capsular polysaccharide conjugates of serotypes present in the PCV13 and seven new serotypes (8, 10A, 11A, 12F, 15B, 22F, and 33F) is currently in development, and a safe profile of PCV20 was confirmed in the 60–64-year age group (22). In our study, 38.2% (13/34) of the tested strains identified five of these seven new serotypes. Newly developed vaccines are expected to prevent the serotype shift of pneumococcal meningitis. We have to carefully monitor the serotype and drug susceptibility of S. pneumoniae strains isolated from patients with meningitis.

This study has some limitations. First, the number of tested strains was relatively low. Second, the tested strains were collected from various hospitals during the study period. Therefore, this study was not a population-based study. Finally, the antimicrobial agents’ data used in each case were collected only for the names of antibiotics. Information on dose and therapeutic dose management could not be collected because of the retrospective nature of the study. National and precise studies on pneumococcal meningitis in Japan are needed in the near future.

In conclusion, our study identified that pneumococcal meningitis in children, after the introduction of PCV13 in Japan, was mainly caused by non-PCV13 serotypes; all cases with sequelae and death were caused by non-PCV13 serotypes. These results suggest that pneumococcal meningitis in children continues to persist and is difficult to control with the current conjugate vaccines. Therefore, we have to carefully monitor the serotype and drug susceptibility of S. pneumoniae strains isolated from patients with meningitis and accordingly reconsider the treatment strategy.

## MATERIALS AND METHODS

### Samples.

We collected S. pneumoniae strains isolated from the cerebrospinal fluid (CSF) or blood from pediatric patients aged less than 15 years with pneumococcal meningitis. All patients were admitted to various hospitals in Japan between November 2013 and January 2020. Pneumococcal meningitis was defined as a systemic infection with positive culture in CSF, or pleocytosis in the CSF with positive blood culture. All S. pneumoniae strains from the pediatric patients residing in Chiba prefecture were analyzed in this study. The population of Chiba prefecture covered approximately 5.0% of the population of Japan. S. pneumoniae strains from the patients residing in other prefectures were analyzed based on the clinician’s request. All isolated strains were analyzed at the Medical Mycology Research Center, Chiba University.

### Capsular serotyping.

Serotypes were determined using the slide agglutination reaction with S. pneumoniae antisera using the Seiken set (Denka Seiken, Tokyo, Japan) and the Quellung reaction using pneumococcal antisera (Statens Serum Institut, Copenhagen, Denmark).

### Antimicrobial susceptibility testing.

Antimicrobial susceptibility testing was performed by broth microdilution according to the guidelines of the Clinical and Laboratory Standards Institute (CLSI) to determine the MIC. The antimicrobial agents used in this study included penicillin G (PCG), ampicillin, CTX, CTRX, MEPM, azithromycin (AZM), clindamycin (CLDM), erythromycin (EM), VCM, LZD, and teicoplanin (TEIC). Antimicrobial susceptibility testing for DAP was performed using the Etest (bioMérieux Japan, Tokyo, Japan) based on the procedure of manufacturer's recommendation (23). MIC breakpoints for CSF S. pneumoniae isolates were determined according to the CLSI criteria: isolates were classified as penicillin-susceptible (MIC ≤0.06 μg/L) and penicillin-resistant (MIC ≥0.12 μg/L).

### Clinical analysis.

We retrospectively analyzed the patients’ clinical features using a questionnaire comprising age, sex, underlying diseases, and vaccine history.

### Statistical analysis.

The chi-squared test was used in the statistical analyses of age, sex, underlying diseases, vaccine history, and serotypes. Some of the serotypes were analyzed using the Fisher exact test. Statistical significance was set at *P* < 0.05. All data were analyzed using JMP Pro version 12 (SAS Institute, Cary, NC, USA).
